# Characteristics of methamphetamine‐related deaths in the United Kingdom, 1997–2024

**DOI:** 10.1111/add.70215

**Published:** 2025-10-15

**Authors:** Emmert Roberts, Rebecca McKetin, Caroline Copeland

**Affiliations:** ^1^ National Addiction Centre King's College London London UK; ^2^ National Drug Alcohol Research Centre (NDARC) University of New South Wales Australia; ^3^ Faculty of Life Sciences and Medicine King's College London London UK

**Keywords:** chemsex, drug‐related deaths, epidemiology, methamphetamine, mortality, toxicology

## Abstract

**Background and aims:**

People who use methamphetamine have a standardised mortality ratio 6.8 times greater than the general population, with several countries reporting concerning increases in methamphetamine‐related mortality over the past decade. Methamphetamine use in the United Kingdom (UK) has been reported as largely confined to communities of men who have sex with men (MSM) with no previous large‐scale studies describing mortality associated with methamphetamine. We aimed to determine trends and case characteristics of methamphetamine‐related deaths in the UK.

**Design:**

Retrospective cohort study.

**Setting:**

Coronial records submitted to the National Programme on Substance Use Mortality (NPSUM) in the UK, 1997–2024.

**Cases:**

Decedents for whom methamphetamine was determined as implicated in death following coronial investigation.

**Measurements:**

Information was available on decedent sociodemographics, characteristics of death and drugs implicated in death.

**Findings:**

136 decedents had methamphetamine implicated in death. The number of deaths per year were observed to be higher over time since the first death recorded in 2006 (2005–2010, 8 deaths; 2011–2015, 24 deaths; 2016–2020, 47 deaths; 2021–2024, 57 deaths). Decedents were predominantly male (*n* = 124, 91%) of White ethnicity (*n* = 68, 50%) with a mean age of 41.5 years (standard deviation 10.4; range 18–71); 77% had a history of substance dependence, 48% of which involved injecting drug use, and 88% had a history of a mental disorder. The median blood methamphetamine concentration detected at post‐mortem was 0.83 mg/l (interquartile range 0.26, 2.5). Multiple drug toxicity was implicated in the majority of cases (*n* = 88, 65%), the most common implicated other drugs being cocaine (*n* = 27, 20%), gamma‐hydroxybutyrate (*n* = 20, 15%), opioids (*n* = 20, 15%), benzodiazepines (*n* = 18, 13%), mephedrone (*n* = 13, 10%) and ketamine (*n* = 12, 8%). Accidental poisoning was the most common direct cause of death (*n* = 89, 65%), with other causes including intentional poisoning, cardiovascular disease, aspiration pneumonia and ischemic bowel disease.

**Conclusions:**

Over the past two decades there appears to have been an increase in the number of methamphetamine‐related deaths in the UK. These deaths largely involve polysubstance use within an overwhelmingly male population with a high prevalence of substance dependence and mental health disorders.

## INTRODUCTION

In recent years multiple countries have reported their highest number of drug‐related deaths since records began [1]. Increasingly this trend has implicated methamphetamine, particularly in areas that have substantial concerns with methamphetamine use and related harms [2, 3]. The standardised mortality ratio (SMR) of people who use methamphetamine has been estimated as 6.8 times higher compared to the general population (SMR = 6.8, 95% CI = 5.3–8.8), largely because of an excess in the number of deaths from poisoning, suicide, homicide, cardiovascular disease and accidental injuries [4]. Understanding the contribution of methamphetamine within national and international drug‐related death trends is important as it may guide policy and practice efforts designed to reduce drug‐related harms.

Within the United Kingdom (UK), population‐level methamphetamine consumption has been historically low, with stimulant use consisting mostly of amphetamine, cocaine and ecstasy [5]. Methamphetamine use is reported to be largely confined to communities of men who have sex with men (MSM) in the context of sexualised drug use or ‘chemsex’ [6]. There has been limited national exploration of methamphetamine‐related deaths despite recent concerns of increasing harm and changing patterns of consumption [6, 7]. To our knowledge, only one previous study has examined methamphetamine‐related deaths in the United Kingdom, reporting on 14 cases where methamphetamine was detected at *post‐mortem* before 2007 [8]. If specific case characteristics and/or particular drug use profiles were found to be associated with fatalities in which methamphetamine was implicated this could lead to the development of targeted interventions or specific harm reduction advice.

To address these gaps, and investigate whether the United Kingdom is experiencing similar trends in methamphetamine‐related deaths to those seen internationally, we aimed to conduct the first epidemiological study to determine: (1) the case characteristics; (2) the circumstances of death; and (3) the number and type of drugs implicated in death among people dying because of methamphetamine‐related causes in the United Kingdom between 1997 and 2024.

## METHODS

This study is reported according to the Strengthening the Reporting of Observational Studies in Epidemiology (STROBE) statement [9], the completed checklist available as Table S1 in the online supplementary material.

### The National Programme on Substance Use Mortality

We conducted a retrospective cohort study using records drawn from the National Programme on Substance Use Mortality (NPSUM), this article containing recycled methods text material from previous NPSUM publications reported in accordance with guidance from the text recycling research project (https://textrecycling.org) [10]. This database contains information on an observational cohort that collects coronial data on deaths at any age related to any psychoactive drug, other than nicotine, caffeine or where the sole substance implicated is alcohol [10]. In total, NPSUM holds records on more than 55 000 deaths with reports received from England, Wales, the Channel Islands and the Isle of Man since 1997. Additional reports were received from the Scottish Crime and Drug Enforcement Agency between 2004 and 2011 and from the General Register Office for Northern Ireland since 2004 with these deaths included within the NPSUM cohort. Deaths are referred to a coroner if the cause of death is unknown, is violent or unnatural, sudden and unexplained, occurred during or following a period of anaesthesia or may have been caused by an industrial disease or poisoning [11]. Coronial files typically include the coroner's decision as to cause of death, statements from witnesses, family and friends, first responders (e.g. police, emergency services), general practitioner (GP) and hospital records, which may include documentation of formal diagnoses and drug use history. Toxicology is discretionally requested by individual coroners, and coronial jurisdictions choose to voluntarily report all deaths within their area to NPSUM if any of the following criteria are met: (1) psychoactive substance(s) are directly implicated in death; (2) an individual has a history of dependence or misuse of drugs; or (3) if controlled drugs are identified at *post‐mortem*. The King's College London (KCL) Biomedical and Health Sciences, Dentistry, Medicine and Natural and Mathematical Sciences Research Ethics Subcommittee (BDM RESC) re‐confirmed in August 2024 that NPSUM does not require Research Ethics Committee (REC) review as all individuals are deceased.

### Case identification

All cases between 1997 and April 2024 were identified from coronial documentation where methamphetamine was coded in the NPSUM database as implicated in the mechanism contributing to death. Only cases in which methamphetamine was implicated were included within this study, cases in which only other amphetamine compounds were implicated were excluded. Final searches were conducted on 17 February 2025.

### Measures

Data on decedent socio‐demographics, mental health history (i.e. a documented history of individual mental health conditions), substance use history (i.e. documented history of substance dependence and injecting status), toxicology, and which individual substances were ultimately deemed implicated in death were extracted from coronial files. All cases additionally recorded the findings concerning the single disease or condition that led directly to death, any antecedent cause(s) and other significant conditions contributory to death. Case files were manually screened to determine if coronial records indicated that death occurred in the context of sexualised drug use.

### Statistical analysis

We generated descriptive statistics for all decedents. Means, SDs and ranges were presented for continuous variables, as well as median and interquartile range (IQR) in skewed distributions. Overall percentages were reported for all binary and categorical variables and the valid percentage (i.e. the percentage of cases where medical history was available) was reported for mental health and substance use variables. All other analyses were conducted in STATA IC version 16. For reference, comparative data for the full NPSUM cohort can be found in the online Supporting information as Tables S2 and S3. The analysis plan was not pre‐registered and the results should be considered exploratory.

## RESULTS

A total of 136 decedents had methamphetamine implicated in their death following drug use in the United Kingdom between 1997 and 2024. The raw number of deaths per year and the population number of deaths per year per 10 000 of all drug‐related deaths in NPSUM can be seen in Figure S1. The number of deaths per year were observed to be higher over time since the first death recorded in 2006 (2005–2010, 8 deaths; 2011–2015, 24 deaths; 2016–2020, 47 deaths; 2021–2024, 57 deaths). The number of deaths per year doubled from a median of 8 deaths per year (IQR = 7–8.5, range = 6–9) in the 4 years before 2020 to a median of 16 deaths per year (IQR = 16–17, range = 16–18) in the 4 years thereafter.

### Socio‐demographics and case characteristics

Socio‐demographic and case characteristics of all decedents can be found in Table 1. Overall decedents were predominantly male (*n* = 124, 91.2%), of White ethnicity (*n* = 68, 50.0%), had a mean age of 41.5 years (SD = 10.4, range = 18–71) and over a quarter were reported as formally unemployed at the time of their death (*n* = 38, 27.9%). Within the 121 cases in which narrative circumstances of death were reported, in only five cases (4.1%) did information submitted to the coroner contain reports that death occurred in the context of sexualised drug use.

**TABLE 1:** Socio‐demographic, case characteristics, circumstances of death and mental health and substance use history of people dying because of methamphetamine‐related causes in the United Kingdom, 1997–2024.

	All, *n* = 136, *n* (%)
Age, mean (y)	41.5 (SD = 10.4; range = 18–71)
Sex	
Female	12 (8.8)
Male	124 (91.2)
Ethnicity	
White	68 (50.0)
Black African	1 (0.7)
Chinese	1 (0.7)
Unknown/not recorded	66 (48.5)
Occupation^a^	
Employed (manual)	22 (16.2)
Employed (non‐manual)	25 (18.4)
Unemployed	38 (27.9)
Student	3 (2.3)
Self‐employed	7 (5.2)
Childcare/houseperson	2 (1.5)
Unknown	9 (6.6)
Year of death	
2005–2010	8 (5.9)
2011–2015	24 (17.7)
2016–2020	47 (34.6)
2020–2024^b^	57 (41.9)
Place of death	
Own place of residence	75 (55.2)
Other residential address	15 (11.0)
Hospital	21 (15.4)
Hotel	8 (5.9)
Other	17 (12.5)
Direct cause of death (ICD‐10 code)	
Poisoning, accidental (X40–X45)	89 (65.4)
Poisoning, intentional (X60–X84)	6 (4.4)
Poisoning, undetermined intent (Y10–Y14)	2 (1.5)
Other	39 (28.6)
Sexualised drug use	
People reportedly engaged in sexualised drug use	5 (3.7)
Mental health^c^	
People with a history of any mental health disorder	30 (88.2)
People with a history of a depressive disorder	16 (47.0)
People with a history of an anxiety disorder	7 (20.6)
Addiction^c^	
People with a history of substance dependence	71 (77.2)
People with an injecting history	13 (52.0)

ICD‐10, International Statistical Classification of Diseases 10th Revision; SD, standard deviation.

^a^ Occupation definitions are based on those used by the United Kingdom Office of National Statistics [16].

^b^ To April 2024.

^c^ Valid percentage reported.

### Characteristics of death and mental health and substance use history

Circumstances of death and mental health and substance use histories of all decedents can be found in Table 1. Poisoning was the predominant disease or condition that was certified as the direct cause of death with coronial determination reporting 89 deaths directly because of accidental poisoning [65.4%; International Statistical Classification of Diseases 10th Revision (ICD‐10) codes, X40–X45], six deaths directly because of intentional self‐poisoning (4.4%; ICD‐10, X60–X84) and two deemed of undetermined intent (1.5%; ICD‐10, Y10–Y14). The remaining 39 deaths reported multiple direct causes including largely cardiovascular (e.g. ischaemic heart disease; ICD‐10, I20–I25) (*n* = 18, 13.2%), respiratory (e.g. aspiration pneumonia; ICD‐10, J69) (*n* = 5, 3.7%) and gastrointestinal (e.g. ischaemic bowel: ICD‐10, K55) (*n* = 4, 2.9%) pathology.

In cases where medical history was available, the majority of decedents had a history of any mental health condition (*n* = 30/34, 88.2%), the two most common documented conditions being a depressive disorder (*n* = 16/34, 47.1%) or an anxiety disorder (*n* = 7/34, 20.6%). The majority of decedents had a history of substance dependence (*n* = 71/92, 77.2%), with almost half of decedents having a history of injecting (*n* = 13/27, 48.1%).

### Number and type of drugs implicated in death

The number and type of drugs implicated in death can be found in Table 2. Where blood levels were available (*n* = 93, 68.4%) the median blood concentration of methamphetamine at death was 0.83 mg/L (IQR = 0.26–2.5, range = 0.008–10.8). The median number of drugs detected at *post‐mortem* was 3 (IQR = 1–5, range = 1–14) with multiple drug toxicity implicated in the majority of cases (*n* = 88, 64.7%). Opioids and benzodiazepines were both implicated in fewer than one in six deaths (*n* = 20, 14.7% and *n* = 18, 13.2%, respectively) with the four most common implicated individual drugs, other than methamphetamine, being cocaine (*n* = 27, 19.9%), gamma‐hydroxybutyrate (GHB) (*n* = 20, 14.7%), mephedrone (*n* = 13, 9.6%) and ketamine (*n* = 12, 8.8%). A minority of decedents had a drug for which they had a prescription implicated in death (*n* = 23, 16.9%).

**TABLE 2:** The number and type of drugs implicated in death as deemed by the coroner among people dying because of methamphetamine‐related causes in the United Kingdom, 1997–2024.

Drug implicated in death^a^	All, *n* = 136, *n* (%)
Methamphetamine	
Median blood concentration (mg/L)	0.83 (IQR = 0.26–2.5; range = 0.008–10.8)
All	
Mean no. of drugs implicated	3.1 (SD = 1.8; range = 1–14)
Median no. of drugs implicated	3 (IQR = 1–5; range = 1–14)
Only methamphetamine implicated	48 (35.3)
Multiple substances implicated	88 (64.7)
Prescribed	
Median no. of prescribed drugs implicated	0 (IQR = 0–1; range = 0–4)
Any prescribed drug implicated	23 (16.9)
Opioids	
Any opioid	20 (14.7)
Heroin	10 (7.4)
Methadone	6 (4.4)
Benzodiazepines	
Any benzodiazepine	18 (13.2)
Diazepam	8 (5.8)
Cocaine	27 (19.9)
GHB^b^	20 (14.7)
Mephedrone	13 (9.6)
Ketamine	12 (8.8)
Alcohol^c^	8 (5.9)

Abbreviations: GBL, gamma‐butyrolactone; GHB, gamma‐hydroxybutyrate; IQR, interquartile range; NPSUM, National Programme on Substance Use Mortality; SD, standard deviation.

^a^ Only those substances implicated in five or more deaths are reported.

^b^ GBL is rapidly metabolised buy the body into GHB.

^c^ Within NPSUM deaths in which alcohol is the only substance detected at *post‐mortem* are not recorded. As such all methamphetamine‐related deaths in which alcohol is implicated in NPSUM have, by definition, multiple substances implicated.

## DISCUSSION

Methamphetamine‐related deaths within the NPSUM cohort have increased in recent years. Although the case characteristics are in some ways typical of UK drug‐related deaths over the same timeframe, including a high proportion of ethnically White decedents and over three‐quarters reporting a mental health disorder and/or substance dependence, atypically over 90% of decedents were male with a high proportion of decedents in active non‐manual employment [7].

Consistent with other studies of methamphetamine‐related deaths, accidental poisoning from multiple drug toxicity was the most common direct cause of death [12]. GHB, mephedrone and ketamine use are over‐represented with fewer than one in six individuals having opioids implicated in death, potentially reflecting previously observed patterns of UK methamphetamine consumption in the context of sexualised drug use [6]. It is, however, notable that in only five cases coronial documentation contained information that the fatality occurred in this context, perhaps highlighting a lack of knowledge, or a lack of inquiry, on the part of professionals submitting coronial documentation and that methamphetamine‐related fatalities can and do occur outside this context. Alcohol and opioids represent the predominant substances used problematically within the United Kingdom, the use of these leading to the overwhelming majority of presentations to addiction services [13]. As such, and despite the increasing trends observed, there is often a limited service offer to people with methamphetamine use disorder and minimal expertise or infrastructure to deliver evidence‐based interventions (e.g. contingency management and emerging pharmacotherapy) [14]. Similarly given the high proportion of decedents in active employment, these individuals may find service opening times restrictive and may be less likely to be engaged with other community or social services, which may identify methamphetamine use and signpost to drug services. Given the burden of substance dependence and mental health disorders we note among decedents, professionals need to be aware of the substantial burden of methamphetamine‐related harm, particularly alongside co‐used other substances and be mindful that harm can occur outside the context of sexualised drug use.

The study has several strengths including the granular examination of substances implicated in death and the large national time frame. There are, however, several important limitations of NPSUM, particularly that explanatory variables of potential interest may either not be available or they may be incomplete. The lack of capture of sexual orientation limited our ability to understand the burden of methamphetamine‐related death within the MSM population or other sub‐populations (e.g. over a third of cases contain no reported ethnicity). NPSUM does not contain coded information on which decedents were accessing evidenced‐based harm reduction or addiction treatment at their time of death, given the proportion of decedents with a history of substance dependence, knowledge of the proportion of people accessing these services would be valuable. Future data linkage to treatment records may, therefore, be a fruitful avenue for further study. Additionally, because of the voluntary submission nature, NPSUM has incomplete coverage of the UK coronial system, with variability of toxicological examination across cases, therefore, some drug‐related deaths will likely have been missed, and some implicated substances not identified [10]. Indeed, only 20 decedents had GHB implicated in death. Given the common co‐use patterns associated with methamphetamine and GHB [typically used as the precursor gamma‐butyrolactone (GBL) in the United Kingdom), coupled with the fact that GHB is not routinely tested for in toxicological screening, deaths relating to methamphetamine and GHB co‐use may be being systematically under identified. Identification of individuals as being involved in sexualised drug use also relies on documentation submitted to the coroner to both contain this information and for it to be accurate [15]. There are likely a number of individuals within NPSUM who were engaged in sexualised drug use where this had either not been established by those submitting coronial evidence, or this was not deemed pertinent to report. As such the numbers reported in this study are likely to represent significantly conservative estimates.

In summary, there have been increases in the number of methamphetamine‐related deaths over the past two decades in the United Kingdom. Polysubstance, GHB, mephedrone and ketamine use are over‐represented within a largely male and employed population with a significant burden of mental health disorders and substance dependence. Monitoring this trend while ensuring addiction services are able to offer evidence‐based interventions for methamphetamine use disorder should be a priority. While bespoke services or support pathways for individuals who have problems with sexualised drug use remain vital, they may not be appropriate for all individuals at risk of methamphetamine‐related harm.

## AUTHOR CONTRIBUTIONS

**Emmert Roberts:** Conceptualization (equal); formal analysis (lead); funding acquisition (lead); investigation (lead); methodology (lead); project administration (lead); resources (equal); software (equal); writing—original draft (lead). **Rebecca McKetin:** Investigation (equal); methodology (equal); supervision (equal); validation (equal); writing—review and editing (equal). **Caroline Copeland:** Conceptualization (equal); data curation (lead); investigation (equal); methodology (equal); project administration (equal); resources (equal); supervision (equal); validation (equal); writing—review and editing (equal).

## DECLARATION OF INTERESTS

All authors have completed the ICJME Unified Competing Interest form (available on request from the corresponding author) and declare: no support from any organisation for the submitted work; no financial relationships with organisations that might have an interest in the submitted work in the previous 3 years, no other relationships or activities that could appear to have influenced the submitted work.

## Supporting information


**Figure S1:** The number of people dying per year because of methamphetamine‐related causes in the United Kingdom, 2006–2024.
**Table S1**: Strengthening the Reporting of Observational Studies in Epidemiology (STROBE) statement checklist [6].
**Table S2**: Percentage of sociodemographic, case characteristics, circumstances of death and mental health and substance use history of people dying due to drug‐related causes in the National Programme on Substance Use Mortality (NPSUM) in the United Kingdom, 1997–2024.
**Table S3**: The percentage and type of drugs implicated in death among people dying due to drug‐related causes in the National Programme on Substance Use Mortality (NPSUM) in the United Kingdom, 1997–2024.

## Data Availability

Data are available subject to institutional approvals.
